# Cross‐Cultural Comparison of Adaptive Behaviour Between British and Brazilian Clinical Samples With Neurodevelopmental Disorders

**DOI:** 10.1111/cch.70098

**Published:** 2025-05-22

**Authors:** Tally Lichtensztejn Tafla, Kate Anne Woodcock, Tatiana Pontrelli Mecca, Maria Cristina Triguero Veloz Teixeira

**Affiliations:** ^1^ Center for Research on Childhood and Adolescence, Human Developmental Sciences Graduate Program Mackenzie Presbyterian University São Paulo São Paulo Brazil; ^2^ Centre for Applied Psychology, School of Psychology and Institute for Mental Health University of Birmingham Birmingham UK; ^3^ Department of Mental Health Santa Casa de São Paulo School of Medical Sciences São Paulo São Paulo Brazil

**Keywords:** ABAS‐3, adaptive functioning, autism, cross‐cultural comparison

## Abstract

Adaptive behaviour deficits are limitations in executing daily activities and difficulties in responding to environmental changes, which impact social participation and independence across contexts. Adaptive behaviour measures can be used to guide interventions for individuals with neurodevelopmental disorders. Cross‐cultural studies can contribute to the understanding of adaptive functioning of neurodivergence across countries.

**Purpose:**

The purpose of this study is to evaluate and compare the adaptive behaviour profiles of children and adolescents with neurodevelopmental condition from different countries.

**Methods:**

Forty‐eight children with an autism spectrum diagnosis were equally separated into country groups (Brazil and the United Kingdom) and ages (5–10 and 11–17 years old) and were evaluated with the Adaptive Behavior Assessment System, 3rd Edition (ABAS‐3), the Parent Form (Ages 5–21), using the raw scores of the questionnaire.

**Results:**

The only scale in which a difference between nationality groups was identified was the self‐direction scale, which evaluates skills needed for independence, responsibility and self‐control, with older Brazilians scoring higher than their British peers in the same age group.

**Conclusion:**

Similar profiles of adaptive functioning in individuals with ASD were found across cultures, with a singular difference in the self‐direction scale. The study's findings shed light on the need for interventions to increase adaptive functioning skills acquisition, regardless of the culture or country in which the individual is.

Adaptive functioning (AF) encompasses a set of skills classified as conceptual, social and practical that are learned and performed by individuals to meet the demands of everyday life (Schalock et al. 2021). Previous studies showed AF impairments in children and adolescents with autism spectrum disorder (ASD) (Tillmann et al. 2019), with commonly lower scores for autistic individuals (Trimarco et al. 2020; Tamm et al. 2022; Chandler et al. 2022; Miniarikova et al. 2023). Several factors can influence the AF of an autistic individual, such as cognitive flexibility (Lei et al. 2022), as well as other cognitive abilities (Tillmann et al. 2019; Hodge et al. 2021), emotional and behavioural problems and other comorbid conditions, such as ADHD (Chandler et al. 2022). AF measures can be used to stablish the disorder's severity level (American Psychiatric Association [APA] 2022), to guide interventions to provide support, monitor their effects and enable better course and prognoses (Nevill et al. 2017).

Given the growing recognition of the need to expand research beyond Western, high‐income populations, cross‐cultural studies have become increasingly important (Broesch et al. 2020). Such studies contribute to a better understanding of how different characteristics may vary across cultural contexts and countries. Examining differences in ASD profiles across regions is a crucial step towards understanding the extent to which cultural factors may influence screening and diagnostic measures (Matson et al. 2017) or whether the core symptoms of the condition are primarily inherent to the diagnosis itself rather than shaped by cultural background.

AF changes according to the context (Schalock et al. 2021). Contextual factors, for example, family interactions, educational involvement and career development, are important to understand similarities and differences in the adaptive behaviour of children and adolescents between countries (Schalock et al. 2021). Observations of children with typical development in a natural context from different cultures and countries reveal similar adaptive and independent behaviours in domestic and social environments. These findings have suggested that countries with higher degrees of cultural similarities use adaptive behaviour standards in a similar way (Oakland et al. 2013). However, relevant questions about differences and similarities in adaptive behaviour in populations with neurodevelopmental disorders remain poorly explored. Exploring these aspects can contribute to a better understanding of how the assessment of adaptive behaviour should be sensitive to the cultural contexts in which children with neurodevelopmental disorders live, for example, children with ASD.

Despite this evidence, there are few studies focusing on cross cultural differences in autistic groups. The lack of studies in this area could be an obstacle to a more accurate analyses of a possible AF pattern of the ASD in different contexts. Although there are numerous studies exploring AF in ASD, there are few studies that specifically focused on identifying AF patterns of ASD across different cultural contexts. The scarcity of such studies limits the ability to provide a more precise and comprehensive analysis (Price et al. 2018). As such, our study aims to fill this gap by contributing to the understanding of how AF in individuals with ASD may manifest in distinct cultural contexts. Therefore, this study aimed to evaluate and compare the adaptive behaviour profiles of children and adolescents diagnosed with ASD in Brazil and the United Kingdom.

**Summary**

- The first cross‐cultural study of adaptive behaviour in neurodevelopmental disorders.
- Brazilian adolescents show higher self‐direction than British peers.
- Adaptive functioning in autism is similar across cultures.
- Self‐direction scale differences are found between older Brazilian and British groups.
- The study supports the need for adaptive skill interventions across all cultures.

## 1 Method

### 1.1 Participants

The study design was qualitative and exploratory, with a nonprobabilistic opportunistic sample, composed of 48 autistic children and adolescents equally separated into two country groups: Brazilian and British. Data collection was conducted in person in Brazil and via telephone call in the United Kingdom. The mean age of the study participants was 10.27 (SD = 2648), with a minimum age of 7 years and a maximum of 17 years, and the majority were male (83%). The sample was separated into two age groups (5–10 and 11–17 years old), because of the large differences in expected developmental milestones according to age groups. Most of both groups was in the 5–11 age group (54% for Brazilian and 62.5% for the British group) (Table 1). The caregivers had access to the Voluntary Informed Consent Form and were asked to indicate whether they agreed to participate in the research.

**TABLE 1:** Sociodemographic characteristics and mental health services used by groups according to ages and countries.

Country	Age group	Speech & language therapy	Occupational therapy	Applied behavioural analysis	Other	Frequency (%)	Gender	
							Girls	Boys
UK	5–10	5	2	0	2	13 (27)	1	12
	11–17	2	2	1	2	11 (23)	2	9
BR	5–10	1	3	4	2	15 (31)	1	14
	11–17	2	0	3	0	9 (19)	4	5
	Total^a^	10	7	8	6	48 (100)	8	40

^a^ The total is 31 because we are only considering the individuals who had access to some kind of intervention (15 Brazilian and 16 British).

The data from the United Kingdom were collected as a part of a wider study on the development of an intervention for emotional outbursts and were collected between 2015 and 2016, and the inclusion criteria was based on parent report of a clinical diagnosis. Children were recruited based on caregivers interested in participating in a caregiver led intervention for emotional outbursts. Brazilian parents were recruited in clinics for mental health services for individuals with ASD. The Brazilian data were collected in 2022, in São Paulo state, and the inclusion criteria was also based on parent report of a clinical diagnosis.

### 1.2 Materials

The Parent 5‐21 Form of The Adaptive Behavior Assessment System (ABAS‐3) has 11 scales distributed among social (leisure and social scales), conceptual (communication, self‐direction and functional academic skills) and practical domains (community use, health and safety, self‐care, school living and work scale). Respondents rate the items indicating whether the individual performs a behaviour independently and, if so, how often it is performed, ranging from 0 to 3 points (Harrison and Oakland 2015).

Sociodemographic questionnaire describes gender, age, medication and mental health services used within the past 6 months.

### 1.3 Data Analysis

The Mann–Whitney test was performed to identify differences in the scores of the different scales according to the nationality of the individuals, in addition to the Kruskal–Wallis' test to compare raw AF scores by each scale according to age groups between the two samples. Based on traditional guidelines (Cohen 1988; Rosenthal and Rubin 2003), the 𝑟 interpretation is as follows: small effect: 𝑟 = 0.1; medium effect: 𝑟 = 0.3; and large effect: 𝑟 = 0.5. Significance level of *p* ≤ 0.05 was adopted. The database for this work can be accessed online (at https://osf.io/2szx5/).

## 2 Results

**FIGURE 1:**
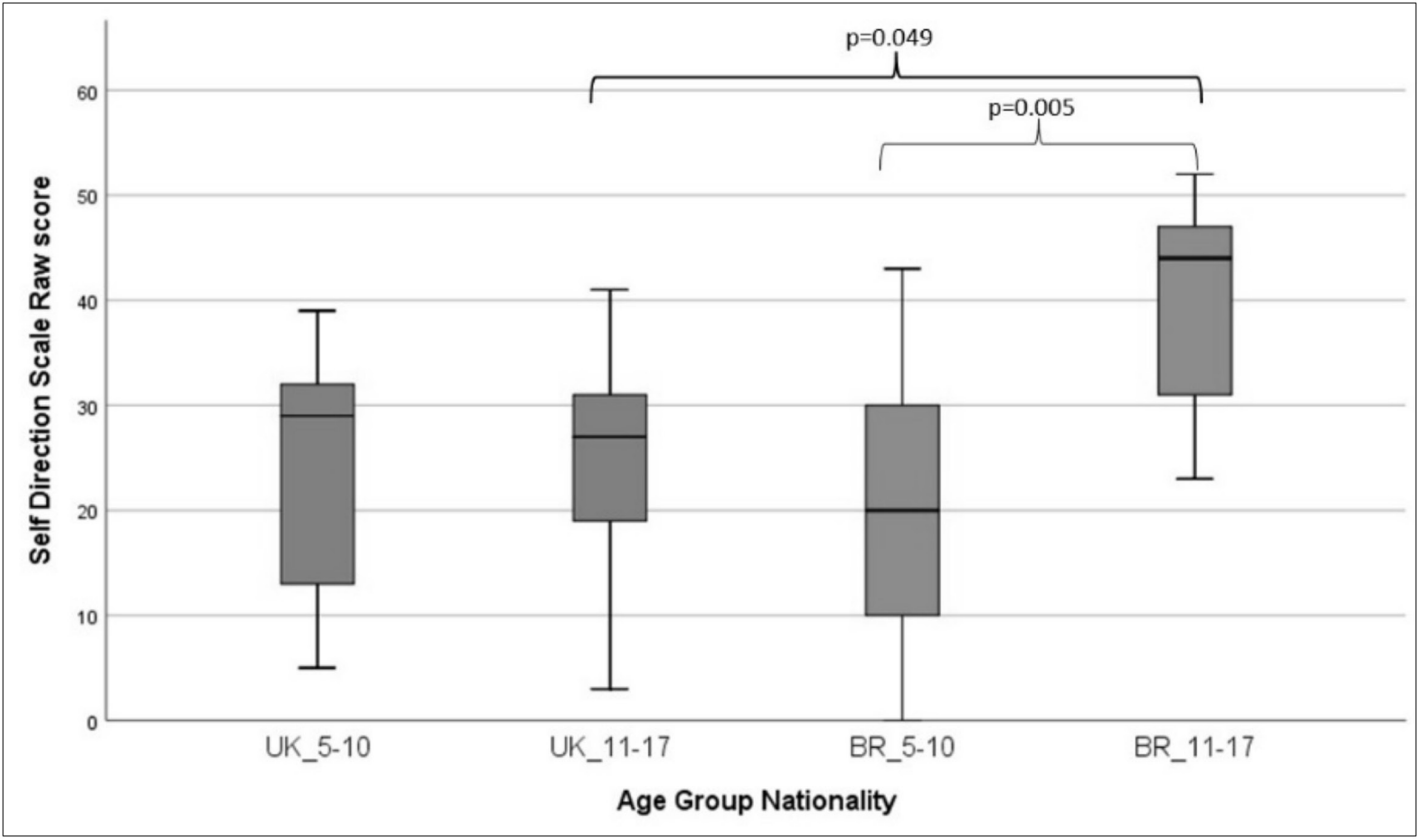
Comparison between age groups and nationality on the self‐direction scale of the ABAS‐3.

The Kruskal–Wallis tests were used to compare the average ranks of the ABAS‐3 scales with the Brazilian and British groups, considering age groups, which showed that there is a difference between age and nationality groups in the self‐direction scale (*H*[3] = 11.968; *p* = 0.007) (Figure 1). Analysis of multiple comparisons (post hoc pair‐wise analyses) made it possible to identify that the differences in the self‐direction scale were between the Brazilian age groups (*z* = −3.331; *p* = 0.005, *r* = 0.480) and between the two nationalities of the older groups (*z* = −2.642; *p* = 0.049, *r* = 0.381), with Brazilians aged 11–17 scoring higher than their British peers in the same age group on the scale. On the Home Living Scale, there was a difference between the Brazilian age groups (*z* = −3.142; *p* = 0.010, *r* = 0.453), with higher scores in the older group. The observed effect size (*r*) was moderate in both cases.

The Mann–Whitney *U* test was also performed to identify differences in the scores of the different scales according only to the nationality of the individuals. However, no significant differences were observed for any of the scales (*p* values were communication = 0.50; community use = 0.95; functional academics = 0.15; home living = 0.96; health and safety = 0.62; leisure = 0.47; self‐care = 0.95; self‐direction = 0.36; and social = 0.78). Therefore, it can be concluded that differences between nationalities were only demonstrated when the groups are divided by age. The database for the analysis is available online (at https://osf.io/2szx5/?view_only=75078808e53a4a0ba95a5ed174a811c4) and can be made available upon reasonable request.

## 3 Discussion

This study aimed to compare the AF profiles of children and adolescents diagnosed with ASD between Brazil and the United Kingdom using the ABAS‐3. Our findings indicated no significant differences in AF scores between the two groups, except for the older group (ages 11–17) at the self‐direction scale, suggesting a similar AF profile across these cultures. This reinforces the idea that AF deficits in individuals with ASD are primarily associated with the condition itself rather than cultural influences on parental perceptions and reporting (Matson et al. 2017).

One key contribution of this study is its challenge to the assumption that individuals with neurodevelopmental conditions from low‐ and middle‐income countries necessarily exhibit lower AF levels. Our results align with previous research (Montenegro et al. 2022) and highlight the importance of considering diagnostic criteria and condition‐specific characteristics rather than assuming cultural disparities in AF outcomes.

We identified difference between countries in self‐direction scale, with Brazilians aged 11–17 scoring higher than their British peers. This scale assesses skills needed for independence, responsibility and self‐control (e.g., making choices, starting, and completing tasks, following a daily routine and following directions) (Harrison and Oakland 2015). These behaviours partially depend on the adequate development of executive functions, which are known to be deficient in individuals with ASD (Craig et al. 2016), and their development is influenced by environmental and cultural factors (Howard et al. 2020).

A hypothesis, supported by the study's findings, is that interventions based on Applied Behavior Analysis (ABA)—more frequent in the Brazilian sample (Araripe et al. 2022)—can stimulate behaviours and skills assessed on the self‐direction scale, which could indicate the higher scores for the Brazilian group. Behaviour regulations, such as inhibition, working memory and planning, are part of the intervention plans of most ABA‐based programmes (Pasqualotto et al. 2021), and ABA interventions have already been described in the literature with potential effects to increase AF (Eckes et al. 2023).

As for the increase in the score observed between age groups only for the Brazilian participants, it is not possible to state that age is the only predictor of the differences found between age groups. However, previous studies results have already verified an increasing trajectory in the daily living skills score over preschool years for children with ASD (di Rezze et al. 2019) and throughout childhood and adolescence (Bal et al. 2015).

It is noteworthy that factors such as level of support and intellectual quotient can influence AF trajectories in individuals with ASD (Tillmann et al. 2019). In our study, we did not investigate these variables, so the British sample may present a different cognitive and clinical profile than the Brazilian sample. This is an explanatory hypothesis for the difference observed between countries about age groups.

The study has limitations. The first is that the IQ of the participants was not evaluated and, therefore, could not be considered in the analysis. Future studies should compare not only AF differences between countries but also intellectual functioning, along with other relevant variables. Additionally, the mental health of parents and their possible beliefs about their children's conditions were not evaluated, which may directly influence their expectations and, consequently, their children's performance. Another limitation of the present study is the uneven gender distribution between groups, particularly the higher proportion of females in the older Brazilian group. Although recent data indicate a narrower diagnostic gap between boys and girls, with approximately 67.7% of individuals with an ASD diagnosis being boys (Global Burden of Disease 2025)—our study reports a ratio of approximately 1 girl to 4.88 boys, which is similar to other global autism prevalence reports (Zeidan et al. 2022). Future cross‐cultural studies of autism that aim to balance gender disparity are recommended. Furthermore, there was a difference in data collection methods: Although data from Brazilian participants were collected in person, data from UK participants were collected via telephone. This discrepancy may have influenced caregivers' responses, and future studies using consistent data collection methods across groups are recommended. Finally, the relatively small sample size, particularly within each subgroup, may have limited the study's power to detect differences beyond those found in the self‐direction scale. Larger sample sizes would allow for more robust conclusions about cross‐cultural differences in adaptive behaviour. Another important consideration is the difference in the timing of data collection. Whereas data from the United Kingdom were collected in 2015 and 2016, data from Brazil were collected in 2022, after the COVID‐19 pandemic. This discrepancy may have influenced the development of certain adaptive skills, particularly those related to autonomy, independence and household responsibilities (dal Pai et al. 2022). Therefore, readers should interpret these results with caution, considering the potential effects of this contextual difference. An additional limitation of this study is the geographic restriction of the Brazilian sample to São Paulo, an urban area. This limits the generalizability of the findings to other regions of Brazil, which may differ in socioeconomic factors and access to resources. Furthermore, the absence of a specific measure of socioeconomic status (SES) means we cannot directly compare the SES of Brazilian participants to that of the UK sample, which could have influenced the results. Future studies should aim to include more diverse regions and consider socioeconomic variables to better understand their potential impact on AF in ASD.

Although we acknowledge that this study is still in its early stages, it provides valuable insights into the role of cultural differences in AF. These initial findings are crucial for advancing the understanding of how cultural context should be considered, particularly when evaluating and supporting children with neurodevelopmental disorders. The present study's findings, in line with the literature, shed light on the need for interventions to increase AF skills acquisition, regardless of the culture or country in which the individual is, to improve autistic children, adolescents and adult mental health outcomes. As such, our study aimed to contribute to the understanding of how AF in individuals with ASD may manifest in distinct cultural contexts.

## Author Contributions

**Tally Lichtensztejn Tafla:** conceptualization, investigation, funding acquisition, writing – original draft, methodology, writing – review and editing, formal analysis, data curation, validation, visualization. **Kate Anne Woodcock:** conceptualization, investigation, funding acquisition, writing – original draft, writing – review and editing. **Tatiana Pontrelli Mecca:** writing – review and editing. **Maria Cristina Triguero Veloz Teixeira:** writing – review and editing, writing – original draft, conceptualization, investigation, funding acquisition, resources, supervision, data curation, project administration, methodology, validation, visualization.

## Ethics Statement

All procedures performed in studies involving human participants were in accordance with the ethical standards of the institutional research committee and with the 1964 Helsinki Declaration and its later amendments or comparable ethical standards. The study was approved by the ethics committee of Mackenzie Presbyterian University under the code number: CAAE: 37319620.6.0000.0084. Informed consent was obtained from all parents or legal guardians.

## Consent to Participate

Informed consent was obtained from caregivers.

## Conflicts of Interest

The authors declare no conflicts of interest.

## Data Availability

The data that support the findings of this study are openly available in OSF (at https://osf.io/2szx5/).
